# A Study Protocol for Increasing Access to Smoking Cessation Treatments for Low-Income Minority Smokers

**DOI:** 10.3389/fpubh.2021.762784

**Published:** 2021-12-02

**Authors:** Alicia K. Matthews, Karriem S. Watson, Cherdsak Duangchan, Alana Steffen, Robert Winn

**Affiliations:** ^1^College of Nursing, University of Illinois at Chicago, Chicago, IL, United States; ^2^University of Illinois Cancer Center, University of Illinois at Chicago, Chicago, IL, United States; ^3^School of Public Health, University of Illinois at Chicago, Chicago, IL, United States; ^4^Massey Cancer Center, Virginia Commonwealth University, Richmond, VA, United States; ^5^School of Medicine, Virginia Commonwealth University, Richmond, VA, United States

**Keywords:** smoking cessation, access to care, social determinants, patient portals, federally qualified health center (FQHC), health disparities

## Abstract

**Background:** Smoking rates among low-income patients are double those of the general population. Access to health care is an essential social determinant of health. Federally qualified health care centers (FQHC) are government-supported and community-based centers to increase access to health care for non-insured and underinsured patients. However, barriers to implementation impact adherence and sustainability of evidence-based smoking cessation within FQHC settings. To address this implementation barrier, our multi-disciplinary team proposes Mi QUIT CARE (Mile Square QUIT Community-Access-Referral-Expansion) to establish the acceptability, feasibility, and capacity of an FQHC system to deliver an evidence-based and multi-level intervention to increase patient engagement with a state tobacco quitline.

**Methods:** A mixed-method approach, rooted in an implementation science framework of RE-AIM (Reach, Effectiveness, Adoption, Implementation, and Maintenance), will be used in this hybrid effectiveness-implementation design. We aim to evaluate the efficacy of a novel delivery system (patient portal) for increasing access to smoking cessation treatment. In preparation for a future randomized clinical trial of Mi QUIT CARE, we will conduct the following developmental research: (1) Examine the burden of tobacco among patient populations served by our partner FQHC, (2) Evaluate among FQHC patients and health care providers, knowledge, attitudes, barriers, and facilitators related to smoking cessation and our intervention components, (3) Evaluate the use of tailored communication strategies and patient navigation to increase patient portal uptake among patients, and (4) To test the acceptability, feasibility, and capacity of the partner FQHC to deliver Mi QUIT CARE.

**Discussion:** This study provides a model for developing and implementing smoking and other health promotion interventions for low-income patients delivered via patient health portals. If successful, the intervention has important implications for addressing a critical social determinant of cancer and other tobacco-related morbidities.

**Trial Registration:** U.S. National Institutes of Health Clinical Trials, NCT04827420, https://clinicaltrials.gov/ct2/show/NCT04827420.

## Introduction

Smoking rates among adults in the United States are at a 50-year low (14.1%) (1). Despite overall declines in prevalence rates, smoking remains elevated among multiple underserved communities. For example, in Chicago, smoking rates among Blacks are significantly higher compared to whites (25.2 vs. 13.2%, respectively) (2). In urban areas like Chicago, smoking rates are also more pronounced among individuals living at or below the federal poverty level (26.8%) (2). Due to historical and current practices of structural racism, including redlining and community divestment, many Blacks in Chicago reside in neighborhoods characterized by concentrated disadvantage, racial segregation, and poor access to health care (3). Indeed, smoking rates in Chicago community areas with the highest poverty and racial segregation range from 22 to 35% of community residents (2). The negative consequences of smoking are well-established, with smoking contributing to a range of life-limiting conditions, including lung cancer, chronic obstructive pulmonary disease, and emphysema (1). In Cook County, where Chicago is located, lung cancer rates among Blacks are substantially higher than whites (116.9 vs. 81.1 per 100,000 for men and 63.3 vs. 54.7 per 10,000 for women) (2). Further, the all-cause morbidity and mortality due to smoking are higher among low-income and Black smokers due to a high prevalence of illnesses exacerbated by smoking (e.g., diabetes) (4). Combined, these inequalities underscore tobacco use as an urgent public health priority for Chicago and similar urban areas across the United States.

Reduced access to smoking cessation treatments, a key social determinant of health, is a persistent driver of smoking-related health inequalities among lower-income and racial/ethnic minority groups. Federally Qualified Health Care Centers (FQHCs) are safety-net clinics that serve low-income and uninsured patients. A recent study found that the overall proportion of tobacco use in FHQCs across multiple states was 25.8% compared to 20.6% in the general population (5). Mile Square Health Center (MSHC) is a network of FQHCs located in the greater Chicago metropolitan area, including Chicago, Rockford, and Cicero, Illinois. MSHC clinics are located in high-poverty neighborhoods with documented inequalities in lung cancer and other smoking-related health inequalities (i.e., asthma). Given the high levels of tobacco use observed among FQHC patients, health system-wide tobacco cessation interventions can potentially improve health inequalities at the patient's level and the surrounding community areas.

### Tobacco Cessation Treatments

In 2000, the U.S. Public Health Service clinical practice guideline, Treating Tobacco Use and Dependence, recommended that providers consistently identify and document patients' tobacco use status and treat tobacco users via the “5As” framework (Ask-Advise-Assess-Assist-Arrange) (6). Although effective, the 5As model is time-consuming and can be challenging to implement in high-volume clinical settings (7). A simplified version of the framework (Ask-Advise-Refer, [AAR]) was subsequently developed (8). When implemented in clinical settings, AAR has demonstrated effectiveness for increasing patient engagement with recommended treatment approaches such as state tobacco quitlines (9–12). State-run tobacco quitlines offer free telephone counseling and nicotine replacement for low-income smokers. The average quit rates among quitline users are 12.7%, increasing to 28.1% when counseling is combined with nicotine replacement (6). Despite the efficacy of provider interventions such as AAR for linking smokers to treatment, these interventions are underutilized in clinical settings.

FQHCs are required to report annually on their implementation and dissemination of evidence-based tobacco cessation per their Uniform Data Set (UDS) guidelines for FQHCs (5). The Centers for Disease Control and Prevention reports that, although 62.7% of outpatient visits included tobacco screening, only 20.9% of current tobacco users received counseling and 7.6% received a prescription for pharmacotherapy during their visit (13). Barriers to provider adherence to AAR practice guidelines are well-documented (i.e., time restraints) (7). As such, innovation in implementing AAR clinical practice guidelines is needed to facilitate the delivery of evidence-based smoking cessation treatments, especially in clinical populations disproportionately burdened by tobacco use.

Advances in electronic health records (EHR) have allowed the delivery of population health interventions in clinical settings. EHRs are a means for systematically obtaining and electronically storing details about a patient's health history, including demographic characteristics, clinical diagnoses, and treatment histories. A key feature of EHR is that they increase the safety and quality of health care services by allowing for the sharing of information among health care providers both within and across health institutions. Using patient health portals is an innovative strategy for proactively offering health promotion information and guidance at the health system level. Patient portals are tied to EHR and are secure online tools specifically designed to help patients access and manage their health history, including communicating with their providers (14). Patient portals allow patients to view a subset of the more extensive health-related information contained in their EHR (e.g., diagnoses, medication lists, appointments). In addition, specific information can be provided to patients via their patient portals outside of a traditional health care visit. Patient health portals can be accessed via computers or internet-enabled smartphones (15, 16). According to the American Hospital Association, 93% of hospitals provide patients access to electric health records (EHRs) *via* patient portals (17). Data from the National Cancer Institute found that 52% of patients reported being offered access to their patient portals by their providers (18). Enrolling in newly available patient portal systems has demonstrated effectiveness in increasing patient-provider communication access to health information and delivering evidence-based preventive services (14, 18).

To date, a small number of randomized controlled trials have used the EHR to identify an entire population of smokers and proactively engage them in treatment (14, 18–21). Proactive engagement can be defined as the systematic targeting of all smokers in a population (e.g., health care system). Proactively calling smokers in the general population to offer free quitline counseling increases quit attempts and cessation rates (22). Several trials have demonstrated the benefit of population-based outreach efforts compared to standard clinical practice on receipt of smoking cessation counseling or medications (range 12.8–14.5 vs. 5.1–7.3%, respectively), and abstinence rates (range 5.3–13.5 vs. 1.1–10.9%, respectively) (20). A few studies have evaluated the reach and feasibility of delivering health promotion interventions *via* patient portals (14, 23). However, few have tested the use of patient portals to offer population-level smoking cessation treatments consistent with the AAR framework that directly links smokers to a state tobacco quitline and does not require trained clinical staff to implement.

Despite the early promise, the potential of patient portals as a health intervention delivery system will be limited by patient enrollment, especially among patients impacted by the digital divide (24). Integrating the promotion of patient portal use into routine primary care practices and offering assistance in enrollment may increase the use of patient portals (25). Patient navigation (PN) is a recognized and evidence-based approach for reducing health inequalities (26) and has been shown to increase patient access to health care services (27). The primary role of the PN is to address patients' informational, emotional, and practical needs associated with accessing health care. A recent meta-analysis of randomized clinical trials of PN interventions demonstrated that, compared to usual care, patients who received PN were significantly more likely to access health screening (OR 2.48, *p* < 0.00001) and to attend a recommended care event (OR 2.55, *p* < 0.01) (27). However, more research is needed to evaluate whether PN can increase patient portal access among patients with low health literacy, racial/ethnic minorities, and patients with limited computer skills; thereby, helping to realize the potential of patient portals for widespread delivery of smoking cessation interventions.

There is strong evidence that health care providers offering screening, brief counseling, and pharmacotherapy reduces tobacco use (6). However, consistent delivery of provider-led tobacco cessation treatments in clinical settings is challenging (28). Our multi-disciplinary team proposes Mi QUIT CARE, an innovative implementation strategy for providing guideline-concordant tobacco treatment in an urban FQHC system to address this implementation barrier. Informed by the socioecological framework (29), my Mi Quit Care includes electronically delivering the AAR brief smoking cessation intervention via the patient portal (8–12). This approach will allow for proactively linking all identified smokers to the state tobacco quitline (30). Further, we will provide patient navigation to reduce barriers to engagement with the patient portal and the state tobacco quitline (26, 27). Patient portals represent a promising strategy for enhancing access to smoking cessation treatments among low-income smokers. However, developmental work is necessary before a full implementation trial to increase this approach's feasibility, acceptability, and cultural appropriateness. As such, the specific aims of this formative study are to:

1. Examine the burden of tobacco use (smoking prevalence) and its influence on pulmonary health inequalities (lung cancer, COPD, and asthma) in the patient populations served by MSHC.
2. To evaluate among MSHC patients and health care providers, knowledge, attitudes, barriers, and facilitators related to smoking cessation, engagement with the tobacco quitline, linkage to the tobacco quitline via a patient health portal, and receipt of patient navigation to facilitate access to the tobacco quitline.
3. To evaluate the use of tailored communication strategies and patient navigation to increase patient portal uptake among patients receiving care at MSHC.
4. To test the acceptability, feasibility, and capacity of a federally qualified health care system to deliver Mi QUIT CARE, an evidence-based and multi-level intervention to increase engagement with the quitline via the patient portal.

## Materials and Methods

### Study Design

**Figure 1:**
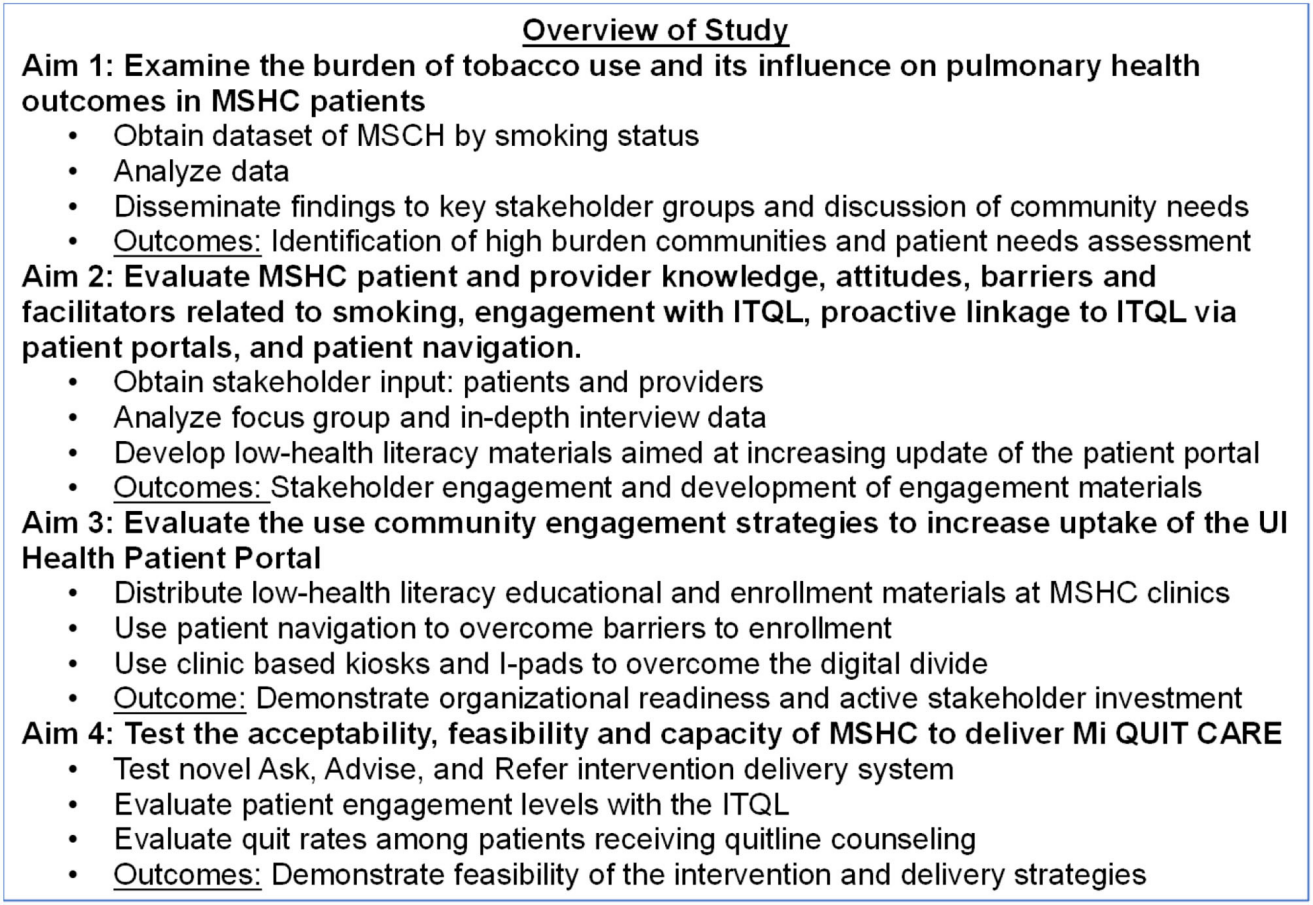
Overview of study design.

Study design and procedures are described below according to study-specific aims (see Figure 1). Qualitative (examining implementation) and quantitative methods (following a randomized controlled trial design) will be used in this hybrid effectiveness-implementation pilot design (31) to evaluate the feasibility and acceptability of a novel delivery system (patient portal) in increasing access to effective smoking cessation treatments. To ensure the scientific rigor and reproducibility of the study, we will use an established evaluation framework, RE-AIM (32). RE-AIM is a planning and evaluation model that addresses five dimensions of the individual- and setting-level outcomes critical to program impact and sustainability: Reach, Effectiveness, Adoption, Implementation, and Maintenance (32). RE-AIM was selected because it is a valuable framework for planning, implementing, and evaluating practice-based interventions to improve external validity.

Further, we will use well-tested data collection methods, training, and supervision to ensure intervention fidelity and biochemical verification of smoking status. The overall study protocol has been approved by the University of Illinois at Chicago Institutional Review Board (Protocol # 2020-0532). Aim 1 of the study was determined to be exempt due to the lack of human subject involvement (Protocol #2020-1621). Aim 2 was determined to be exempt from IRB approval due to minimal risk (Protocol #2021-0578). However, informed consent will be obtained from all study participants in this aim. Each aim of the study will be reviewed and approved by the IRB committee and informed consent obtained from participants in each of the aims determined by the IRB to represent human subjects research. The study funding period and study timeline will take place between June 2020-May 2023.

### Theoretical Model

**Figure 2:**
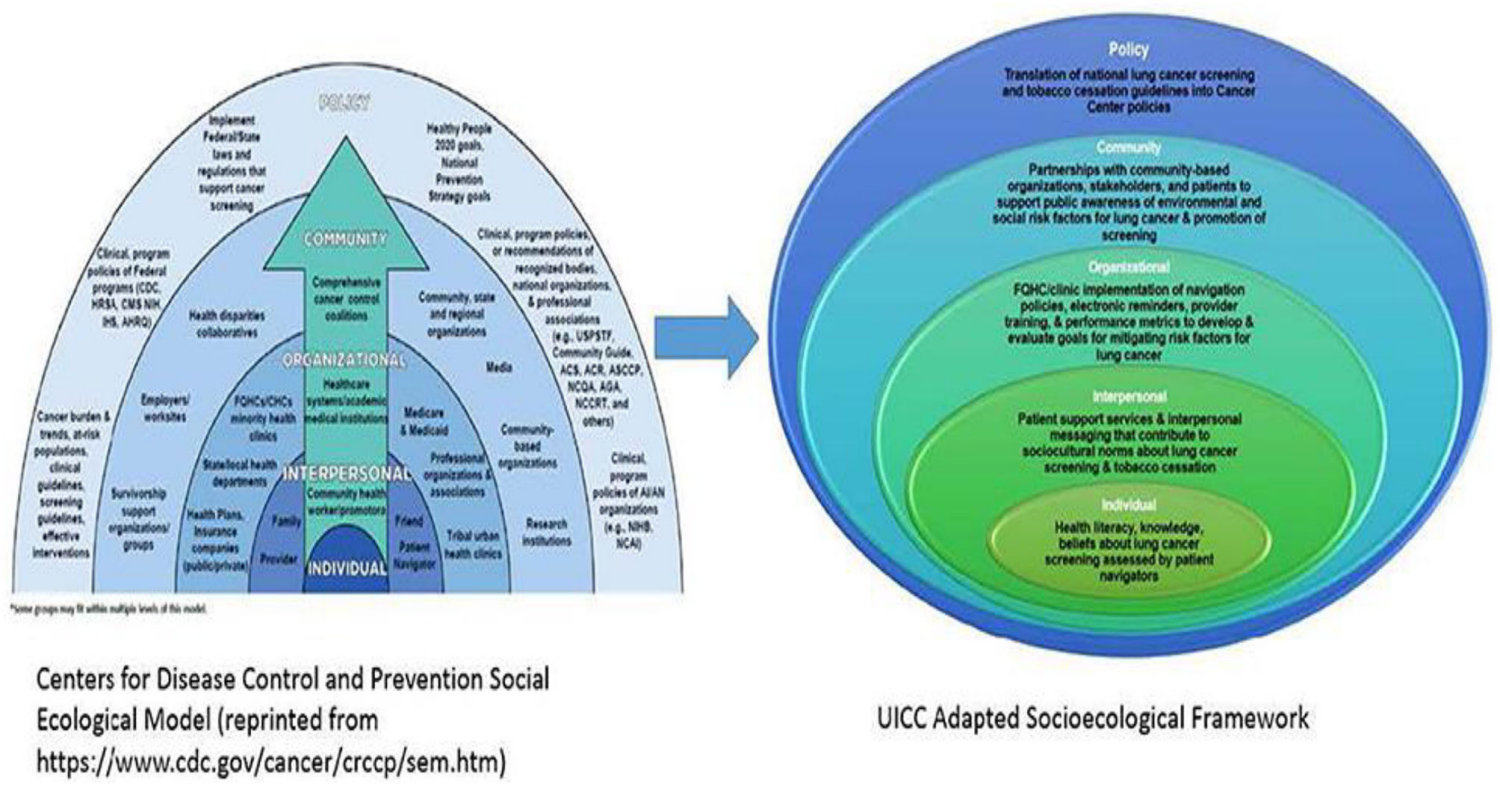
UICC adapted socioecological framework.

Mi QUIT CARE is informed by the Centers for Disease Control's Socioecological Model (SEM) (29) and cognitive-behavioral theories (33). The University of Illinois (UI) Cancer Center and MSHC have experience conducting multi-level interventions to reduce cancer-related disparities (34). Guided by the SEM (29), the UI Cancer Center has proposed a new pathway in reducing pulmonary health inequalities. At the *individual level* and consistent with prior research, cognitive-behavioral models of behavior change (i.e., Theory of Planned Behavior) (33) will be used to understand attitudes toward smoking cessation among patients at FQHCs. *Interpersonal interventions* will include patient navigators to support patient uptake of the patient portals and address barriers to receiving evidence-based tobacco cessation treatments. We will evaluate a system-wide smoking cessation intervention delivered via a patient health portal at the organizational level. *Community-level support* is fostered through ongoing partnerships with organizations like the American Lung Association and the Illinois Tobacco Quitline. Lastly, at the *policy level*, the UI Cancer Center acknowledges the role of policy in improving pulmonary health outcomes, such as the regulation of flavored tobacco products, including mentholated brands (35, 36) (see Figure 2).

### Setting

This multi-level intervention will be delivered via the University of Illinois Hospital and Health Sciences System (UI Health) Patient Portal, a cost-effective and sustainable intervention delivery system. Mi QUIT CARE will be conducted in six Mile Square Health Center (MSHC) clinics, a network of 13 community-located FQHCs, including school-based health centers. MSHC is co-owned and operated by UI Health. MSHC serves communities experiencing a high burden of pulmonary health morbidity and mortality associated with tobacco use. The majority of MSHC patients are Black (74%), live at or below the federal poverty level (98%), and are insured through Medicaid (55%, 10% are uninsured). MSHC has the infrastructure to complete the study and monitor the impact of the intervention on their patients' smoking and pulmonary health status over time. Metrics demonstrating implementation readiness include: (1) consistent assessment and documentation of smoking status in the electronic health record by providers; (2) current availability of a system-wide patient portal that can be used to communicate with patients regarding their care; (3) an established collaboration with the Illinois Tobacco Quitline as part of an existing smoking cessation program at MSHC, and (4) an existing patient navigation program for smoking cessation and lung cancer screening.

### Stakeholder Engagement

Community engagement is essential to the development of effective interventions. As shown in Table 1, we have assembled a diverse community advisory board representing multiple engagement levels across the socioecological model (29). Engaging community stakeholders is essential to developing tailored tobacco cessation interventions for at-risk populations (37). Specifically, community engagement will help to ensure: (a) the smoking cessation intervention is informed by and responsive to stakeholder needs; (b) the implementation of evidence-based interventions that align with patient preference and clinic culture; (c) the sustainability and scalability due to iterative stakeholder input; and (d) broad dissemination of findings to local, regional and national organizations (37). The community advisory board will meet quarterly throughout the project and will help to ensure the appropriateness of methods used.

**Table 1:** Mi CARE QUIT community advisory board.

**Level of engagement**	**Stakeholder name or entity**
Policy	American Lung Association Illinois Tobacco Quitline Chicago Department of Public Health American Cancer Society
Community	American Lung Association Illinois Tobacco Quitline Chicago Department of Public Health
Organizational	Providers at MSHC American Lung Association Illinois Tobacco Quitline Chicago Department of Public Health American Cancer Society
Interpersonal	Patient Navigators Implementation and Dissemination Specialist Providers
Individual	Current and former smokers who are patients at MSCH

### Study Procedures

Below is a description of study procedures, separated by study aims.

*Aim 1: Examine the burden of tobacco use (smoking prevalence) and its influence on pulmonary health inequalities (lung cancer, COPD, and asthma) in the patient populations served by MSHC*.

Patient-level data from the electronic health records (EHR) of all six MSHC locations will be analyzed to evaluate the burden of tobacco use in our patient population. MSHC utilizes the epic platform for their EHR and can extract de-identified patient-level data. Eligibility criteria for inclusion in the analyses include (1) age 18 years and older, (2) a patient at one of the six participating MSHC clinics, and (3) having received care at MSHC within the past 2 years. Patient-level demographic data include age, gender, race/ethnicity, education, income status, relationship status, insurance type, and zip code. Smoking data include current smoking status (current, former, never). Smoking-related lung conditions to be examined include a diagnosis of lung cancer, COPD, and asthma. Chronic health conditions are exacerbated by smoking, including HIV infection, high blood pressure, cardiovascular disease, stroke, asthma, and diabetes. The data manager will prepare a request for the data sets, and the study biostatistician will analyze data to characterize the burden of tobacco use on pulmonary health outcomes. Prevalence, comparisons across clinic locations, and associations (e.g., race/ethnicity, gender, age) will be examined. Data from this aim of the study will allow us to establish a baseline level of smoking among the patient population and the presence of smoking-related comorbidities among patients who smoke. These data will serve as secondary endpoints in tracking the progress of our tobacco cessation intervention. No human subjects are involved in this aim.

*Aim 2: Evaluate among MSHC patients and providers, knowledge, attitudes, barriers, and facilitators related to smoking cessation, engagement with the tobacco quitline, linkage to the tobacco quitline via a patient health portal, and receipt of patient navigation to facilitate access to the tobacco quitline*.

A qualitative design will obtain stakeholder input on the interventions to test Mi QUIT CARE. Five focus groups (*N* = 50) will be conducted with current smokers, and in-depth interviews will be conducted with providers at MSHC (*N* = 24). The goals of the qualitative interviews will be to understand knowledge, attitudes, beliefs, and barriers related to (1) smoking cessation, (2) engagement with the quitline, (3) linkage to the quitline via the UI Health Patient Portal, and (4) the acceptability of patient navigation to facilitate enrollment in the patient portal and address barriers to engaging with the quitline. Eligibility criteria for focus groups include: (1) aged 18 years and older, (2) current smoker, (3) English speaking, and (4) ability to provide informed consent. Eligibility criteria for providers include: (1) employed at MSHC and (2) providing primary care for adults. All study participants will be recruited from MSHC via posted flyers and clinic-based recruitment activities conducted by trained research assistants.

Focus groups (90 min) and individual interviews (45 min) will be conducted according to standardized methodology, including using trained moderators, a moderator's guide, post-session debriefings, and a review of transcribed audiotapes (38). The moderator's guide for the focus groups and in-depth interviews will be developed based on cognitive-behavioral models of health behavior change. Questions will include knowledge, attitudes, perceived social norms, perceived risks and benefits, self-efficacy, and barriers and facilitators regarding receipt of smoking cessation treatment and the use of the patient portal. Focus group participants will also complete a brief demographic survey. Interviews will be analyzed according to the methods of framework analysis (39). We expect to reach saturation with the proposed sample sizes based on our prior experience (40–43). NVivo will be used for qualitative data management and analyses. First, focus groups and in-depth interviews will be analyzed based on study questions and additional sub-themes identified. These design and analytic approaches are appropriate for applied research (39). The information obtained from patients and providers will help us understand initial attitudes and opinions about the intervention approaches and help refine intervention-related strategies.

Based on focus group data, we will partner with health literacy experts to tailor project informational materials to the needs of patients with low levels of health and technology literacy. Informational materials will include information about enrollment and use of the patient portal, communication from MSHC providers advising all current smokers to make a quit attempt, smoking cessation educational pamphlets, and a description of the tobacco quitline. Materials will be tailored to the needs of low-income patients in terms of language, health literacy, and health beliefs. In addition, tailoring of patient educational materials will be on Kreuter's methods for cultural tailoring (38) and will include (a) peripheral (images, etc. salient to smokers); (b) evidential (cancer rates specific to smoking); (c) linguistic (language and terms used by group); (d) constituent-involving (involving diverse populations of smokers); and (e) sociocultural tailoring (including cultural beliefs). After tailoring materials, a new sample of smokers (*N* = 25) and providers (*N* = 10) will be recruited based on the above eligibility criteria. In-depth interviews will collect data on the usability, acceptability, and comprehension of tailored educational materials. A trained research assistant will review materials with individual participants (30 min). The talk-aloud approach (44) will be used to obtain users' feedback. All sessions will be audiotaped, and information reviewed to make suggested changes and to finalize educational materials. All participants will receive a stipend. This aim was deemed exempt from IRB approval due to the low potential risk for participant harm. However, standard information materials will be provided and informed consent obtained before data collection. The information obtained from patients and providers will support the development of the patient portal strategies to increase the intervention's usability, acceptability, and cultural appropriateness.

*Aim 3: Evaluate the use of community engagement strategies to increase uptake of the Patient Portal*.

The utility of patient health portals as a health promotion delivery system will be limited if uptake is low among patients in FQHC who may have issues with general literacy, health literacy, and technology-based literacy levels. In this aim, we will evaluate a tailored multi-modal educational campaign to increase the enrollment of MSHC patients in the UI Health Patient Portal. Currently, only 1% of MSHC patients are enrolled in the patient portal. The goal is to increase enrollment to 40% across the 3 years of the developmental trial to demonstrate the feasibility of delivering Mi QUIT CARE *via* the patient portal. The educational campaign will occur in three MSHC locations (Main, South Shore, and Englewood). The remaining three locations (Back-of-the-Yards, Cicero, and Humboldt Park) will serve as waitlist controls. The following strategies will be used to increase enrollment: (1) branding of the outreach initiative, “*My UI Health”*; (2) conducting a clinic campaign using materials tailored to low health literacy populations; (3) offering written provider recommendation and enrollment information during all clinical encounters; (4) mailing informational letters to all MSHC patients that includes enrollment instructions; and (5) offering onsite enrollment assistance provided by patient navigators. Trained patient navigators will assist clinic patients in enrolling in the patient portal *via* kiosks and secure iPads. After the data collection phase, the patient enrollment campaign will be conducted at the three waitlist control clinics.

Study investigators will first evaluate patient portal enrollment considering all patients aged 18 and older who have had an office visit during the previous 24-month period to establish a baseline comparator. During the campaign, enrollment rates will be examined for each clinic to monitor increases in enrollment in the patient portal during the campaign period. We will compare enrollment rates for the intervention clinics vs. the control clinics. Further, we will evaluate whether additional targeted outreach efforts are needed for some patient groups by analyzing whether demographic factors (age, education, gender, race/ethnicity, last provider visit) are associated with portal enrollment. In addition, we will collect reasons for refusal among patients approached in the clinics by patient navigators. Data on enrollment will establish the feasibility of population-based engagement of health promotion interventions via the patient portal. Informed consent will not be required as this aim is part of a quality improvement project at the clinic to increase access to the patient portal. All analysis of portal uptake will be based on de-identified data.

*Aim 4: Test the acceptability, feasibility, and capacity of MSHC FQHC to deliver Mi QUIT CARE*.

We propose a Hybrid Type I effectiveness-implementation design grounded in RE-AIM (32). Hybrid I types are appropriate for evaluating outcomes associated with clinical intervention and implementation strategies (45). Led by our informatics team, our web application development will be spread across three phases: design, production, and testing/deployment. During the design phase of the study, our technical teams will work closely to refine and document all system specifications and requirements. System logic/rules will be generated. Wireframes, storyboards, interface mock-ups, schematics, and database designs will be produced. In the production phase, the technical team will build the application's site and database. The process will conclude with the testing and deployment phase in which the study and technical teams pass through multiple cycles of quality assurance. After the system is deployed to the live environment, the study and technical teams will stay engaged over the project's lifespan.

All English-speaking adult patients at three MSHC locations (Main, South Shore, and Englewood) who smoke and are enrolled in the UI Health Patient Portal will be eligible for this pilot test. An equal number of patients (total *N* = 100) will be randomly selected from each location for the feasibility trial using a software program developed by programmers at the University of Illinois at Chicago. Human subject approval will be obtained before data collection, and formal informed consent obtained from participants.

**Figure 3:**
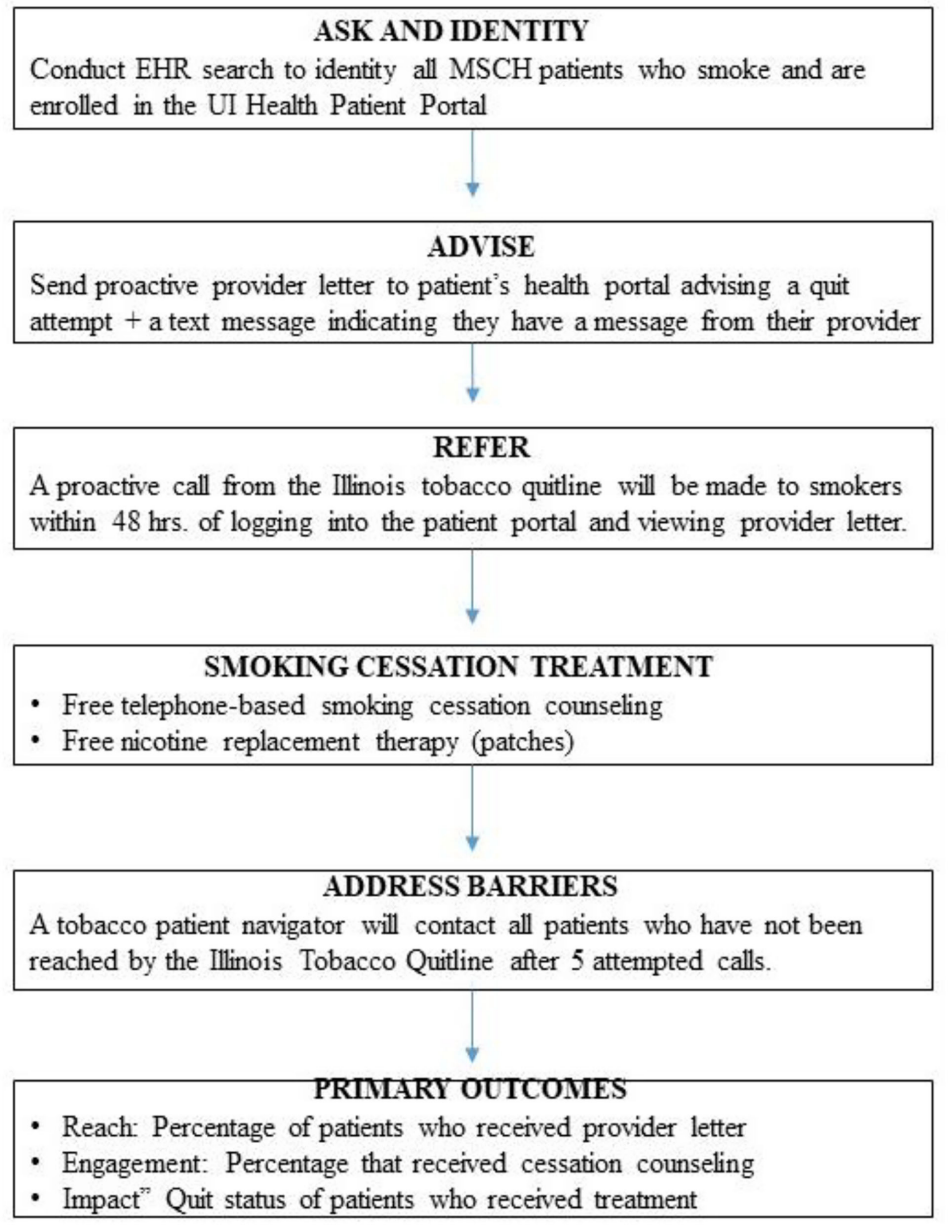
Overview of the brief smoking cessation intervention.

Figure 3 displays an overview of the activities to be conducted in Aim 4 of the study. The intervention will be based on the 3 A's framework (Ask-Advise-Refer, [AAR]) (9, 11, 12). In the first phase of this aim, we will *Ask and Identity, All Smokers*. Project staff will review the electronic health record of the six participating clinic locations to identify all current smokers. Any patient without documented smoking status will be flagged for input during the subsequent clinical encounter. Next, we will *Advise a Quit Attempt*. A random selection of all smokers enrolled in the patient portal will be sent a signed letter from their provider via the portal. The letter will describe health risks associated with smoking, explain the availability of free treatments, strongly encourage the patient to make a quit attempt, and inform them that they will be receiving a call from the Illinois Tobacco Quitline. Patients will be informed that the ITQL will provide free smoking cessation counseling and nicotine replacement therapy (nicotine patches). An automated text or email message with a hyperlink to the portal login page will be generated and sent to patients to alert them of the message from their providers.

#### Refer

The provider letter will describe the benefits of smoking cessation, advise the patient to quit smoking, and notify them they will receive a call from the ITQL within the next 48 h. Once the patient views the electronic provider letter via the patient portal, an automated referral will be sent to the ITQL with the patient's name, phone number, and unique ID number. Patients will also be allowed to opt-out of the treatment engagement call.

#### Treatment

A trained tobacco quitline counselor will call patients. Once reached, quitline counselors will assess the patient's readiness to quit smoking and provide an appropriate treatment plan. If interested in making a quit attempt, quitline counselors will provide smokers with an overview of the program, obtain cessation goals, recommend a treatment approach (counseling only or counseling + nicotine replacement therapy), and schedule the next session. The ITQL counseling program is based on the Freedom from Smoking (46, 47) program. It offers up to 6 weeks of free nicotine replacement (patches) to those 18 and older, medically eligible, and uninsured or on Medicaid. The American Lung Association operates the Illinois Tobacco Quit Line. Certified counselors speak both English and Spanish and have expertise in the treatment of diverse smokers.

#### Address Barriers

Quitline counselors will make 5 attempts to reach the patient before referring the case to the MiQuit Care patient navigator. Trained patient navigators will call non-responders to encourage them to make a quit attempt and engage with the tobacco quitline. A standardized assessment of the patient's interest in smoking cessation and barriers to engagement with the quitline will be conducted. Smokers who are not interested in quitting will receive brief motivational counseling and be advised to make a quit attempt soon. Smokers interested in quitting smoking will be referred to the quitline by the patient navigator using the patient portal. MSHC has an existing lung health navigation program for smoking cessation and lung cancer screening. Study investigators will oversee the training and ongoing supervision of existing lung health patient navigators to ensure fidelity to the treatment protocol.

### Data Analysis

Consistent with RE-AIM (32), our primary outcomes will include Reach and Impact (Reach x Efficacy). We will obtain feasibility data from the patient portal. First, we will assess which patients received (opened) an electronically generated message from their providers advising them to make a quit attempt, describing free stop smoking services from the ITQL, and informing them about an upcoming call from the ITQL. All patients will be allowed to opt into the proactive ITQL counselor call. We will evaluate what percentage of patients opt-in to treatment and the demographic factors associated with the decision to accept linkage to the ITQL. Next, we will assess the percentage of patients who opted-in to treatment and were subsequently reached by the ITQL. Finally, we will evaluate the percentage of patients who engaged in stop smoking treatment (participated in more than 1 counseling call) and their quit outcomes. Patient receipt of treatment and self-reported quit rates will be obtained from the ITQL. The ITQL will provide a monthly report on patient engagement and quit rates. Statistical analyses (multivariable logistic regression models) will be conducted to determine demographic factors (age, race, gender, clinic) associated with receipt of the provider message and advice to quit smoking, receipt of treatment by the ITQL, and quit rates. One of our smoking cessation patient navigators will contact patients who opted-in to linkage to the ITQL and who were not reached by an ITQL counselor. Patient navigators will record barriers to quitline engagement that will be qualitatively analyzed. Data from this aim will refine implementation strategies and procedures in preparation for a fully powered randomized controlled trial.

## Discussion

The purpose of this paper is to describe the engagement and developmental protocol for an NIH-funded research study aimed at increasing access to smoking cessation treatments for FQHC patients. Specifically, we aim to determine the feasibility, acceptability, and capacity of an FQHC system to deliver evidence-based smoking cessation treatments to smokers using a patient health portal. Increasing access to evidence-based and cost-effective smoking cessation treatments is a national priority for reducing pulmonary health inequalities among highly vulnerable patients. Federally qualified health care systems represent a mechanism for addressing the health care needs of low-income and under-insured individuals and communities. However, given the complexity of patient healthcare needs, time for health promotion counseling within the confines of the typical clinical appointment is limited. As such, innovations are needed to provide cost-effective and system-wide approaches to supporting patients in making health-related behavioral changes. Additionally, per their uniform data system requirements, FQHCs are required to report annually on their implementation and uptake of evidence-based tobacco cessation interventions.

Patient portals are increasingly available across various health care systems and are being used to improve patient-provider communication and health-related information. Furthermore, researchers are investigating the use of patient portals to deliver evidence-based health promotion interventions across a range of health promotion behaviors (i.e., diabetes self-management). As has been the case with a variety of health care innovations, low-income, and other marginalized patient populations may not have access to the accompanying benefits of patient health portals due to access barriers, including low literacy levels. Patient engagement approaches in the forms of advisory boards, qualitative studies with patients and providers, further tailoring health information to the needs of patients with low health literacy, and the identification of groups in need of additional assistance in the form of patient navigation and other supportive resources can help to overcome access barriers associated with health-related technologies. Equally important is implementing/dissemination approaches to expand provider and system-level bandwidth to provide patients with needed health promotion interventions, including smoking cessation.

### Limitations

While this study has several strengths and contributes to existing gaps in the literature, we also acknowledge several limitations. First, the sample is drawn from a single FQHC system in a single geographical location. As such, additional research should be conducted with FQHC systems in other geographic areas. Although the percentage of FQHC with access to patient portals is growing, not all locations have the capacity currently, which could lower the impact of the intervention. Finally, patient navigators play an essential role in reducing health disparities. However, not all locations may have them as a part of the established clinic workforce.

## Conclusions

Guided by the RE-AIM framework (32), our proposed study aims to conduct the developmental work necessary to evaluate Mi Quit Care's efficacy and implementation endpoints fully. Study findings from our developmental aims will provide initial data on Reach and Effectiveness. Following completion of this current study, we will conduct a fully-powered randomized clinical trial in which we will confirm the research and efficacy of the study as well as implementation outcomes (Adoption, Implementation, and Maintenance). Further, this study has the potential to develop and deploy evidence-based interventions for FQHCs that are required per their UDE mandates to implement evidence-based tobacco cessation interventions. Combined, the current and proposed future study have the potential to shape knowledge and future research on the feasibility of using patient health portals to deliver smoking cessation to high-risk patient populations receiving treatments in safety-net health centers.

## Ethics Statement

The overall study protocol has been approved by the University of Illinois at Chicago Institutional Review Board (Protocol # 2020-0532) as a Center Grant. No human subjects may be recruited or enrolled, or their records, data, or biospecimens accessed or analyzed, under this protocol. Any human subject research supported by this Center Grant will require a separate application to the UIC IRB. Aim 1 of the study was determined to be exempt due to the lack of human subject involvement (Protocol #2020-1621). Aim 2 was determined to be exempt from IRB approval due to minimal risk (Protocol #2021-0578). However, standardized procedures for obtaining informed verbal consent will be obtained from all study participants in Aim 2. Research activities associated with Aims 3 and 4 have not just been started. However, prior to the initiation of research related to these two aims, the study protocols will be reviewed and approved by the IRB committee and informed written consent obtained from participants in each of the aims determined by the IRB to represent human subjects research.

## Author Contributions

AM, KW, and RW are the multiple principal investigators and designed and drafted the study protocol. AS contributed to the study design and analytic plan. CD contributed to the description of the study protocols and intervention components. All authors contributed to the overall design of the study and the preparation of the protocol manuscript, read, and approved the final manuscript.

## Funding

Funding for this study was provided by a grant from the National Heart Lung and Blood Institute of the National Institutes of Health **(**UG3/UH3 HL151302, Matthews/Watson/Winn, MPI).

## Conflict of Interest

The authors declare that the research was conducted in the absence of any commercial or financial relationships that could be construed as a potential conflict of interest.

## Publisher's Note

All claims expressed in this article are solely those of the authors and do not necessarily represent those of their affiliated organizations, or those of the publisher, the editors and the reviewers. Any product that may be evaluated in this article, or claim that may be made by its manufacturer, is not guaranteed or endorsed by the publisher.

## Abbreviations

AA: African Americans
AAR: Ask-Advise-Refer
ALA: American Lung Association
HER: electric health records
FQHC: Federally Qualified Health Care Centers
ITQL: Illinois Tobacco Quitline
LHL: low-health literacy
Mi QUIT CARE: Mile Square QUIT Community-Access-Referral-Expansion
MSHC: Mile Square Health Center
PN: patient navigation
SEM: socio-ecological model
UI: University of Illinois
UI Health: The University of Illinois Hospital and Health Sciences System.
