# Corrigendum: A Study Protocol for Increasing Access to Smoking Cessation Treatments for Low-Income Minority Smokers

**DOI:** 10.3389/fpubh.2022.863190

**Published:** 2022-02-22

**Authors:** Alicia K. Matthews, Karriem S. Watson, Cherdsak Duangchan, Alana Steffen, Robert Winn

**Affiliations:** ^1^College of Nursing, University of Illinois at Chicago, Chicago, IL, United States; ^2^University of Illinois Cancer Center, University of Illinois at Chicago, Chicago, IL, United States; ^3^School of Public Health, University of Illinois at Chicago, Chicago, IL, United States; ^4^Massey Cancer Center, Virginia Commonwealth University, Richmond, VA, United States; ^5^School of Medicine, Virginia Commonwealth University, Richmond, VA, United States

**Keywords:** smoking cessation, access to care, social determinants, patient portals, federally qualified health center (FQHC), health disparities

In the published article, an author name was incorrectly written as “Cherdsak Duang.” The correct spelling is “Cherdsak Duangchan.”

In the published article, there was an error in affiliation 1. Instead of “College of Nursing, The University of Illinois Chicago, Chicago, IL, United States” it should be “College of Nursing, University of Illinois at Chicago, Chicago, IL, United States.”

In the published article, there was an error in affiliation 2. Instead of “University of Illinois Cancer Center, The University of Illinois Chicago, Chicago, IL, United States” it should be “University of Illinois Cancer Center, University of Illinois at Chicago, Chicago, IL, United States.”

In the published article, there was an error in affiliation 3. Instead of “School of Public Health, The University of Illinois Chicago, Chicago, IL, United States”, it should be “School of Public Health, University of Illinois at Chicago, Chicago, IL, United States.”

The authors apologize for these errors and affirm that they do not change the scientific conclusions of the article in any way. The original article has been updated.

## Publisher's Note

All claims expressed in this article are solely those of the authors and do not necessarily represent those of their affiliated organizations, or those of the publisher, the editors and the reviewers. Any product that may be evaluated in this article, or claim that may be made by its manufacturer, is not guaranteed or endorsed by the publisher.

